# Metastatic Merkel cell carcinoma response to nivolumab

**DOI:** 10.1186/s40425-016-0186-1

**Published:** 2016-11-15

**Authors:** Frances M. Walocko, Benjamin Y. Scheier, Paul W. Harms, Leslie A. Fecher, Christopher D. Lao

**Affiliations:** 1 University of Michigan Medical School, Ann Arbor, MI USA; 2 Division of Hematology/Oncology, Department of Internal Medicine, University of Michigan, C451 Med Inn, 1500 East Medical Center Drive, Ann Arbor, MI 48109 USA; 3 Department of Pathology, University of Michigan, Ann Arbor, MI USA; 4 Department of Dermatology, University of Michigan, Ann Arbor, MI USA

**Keywords:** Merkel cell carcinoma, Nivolumab, PD-L1, Immunotherapy, Polyomavirus, Case report

## Abstract

**Background:**

Merkel cell carcinoma (MCC) is an aggressive cutaneous neuroendocrine malignancy with limited treatment options. Several lines of evidence support the programmed death-1/programmed death-ligand 1 (PD-1/PD-L1) axis as a likely contributor to immune evasion in MCC.

**Case presentation:**

We report a case of a patient with metastatic MCC with a significant and durable response to nivolumab, a humanized IgG4 monoclonal anti-PD-1 antibody.

**Conclusion:**

Immunotherapy with PD-1/PD-L1 inhibitors has become a rational and promising treatment option for MCC in the advanced or metastatic disease. Clinical trials are currently in progress to further evaluate these novel therapeutic agents.

## Background

Merkel cell carcinoma (MCC) is a rare and aggressive cutaneous neuroendocrine malignancy with an annual incidence rate of 0.6 per 100,000 persons [1]. MCC is frequently diagnosed in the elderly in areas of sun-exposed skin and remains a challenging disease to treat. It has a high frequency of local recurrence (30 %) compared to melanoma (3.8 %), and more than 40 % of patients eventually develop distant metastatic disease [2, 3]. Early stage disease is typically managed by surgical excision with or without radiotherapy [4]. Adjuvant radiotherapy may improve locoregional control for some tumors, but it is unclear if it impacts survival [5, 6]. Patients with unresectable locally advanced or metastatic disease are most often treated with chemotherapy [7]. While there are currently no Food and Drug Administration (FDA) approved therapies for advanced MCC, platinum in combination with etoposide has been the standard for advanced stage MCC based on data from small cell lung carcinoma. Cyclophosphamide, doxorubicin and vincristine have also been tried with variable response rates [8]. Unfortunately, responses to chemotherapy are not durable and have not clearly demonstrated a survival advantage [9]. New approaches for the disease are needed.

## Case presentation

**Fig. 1:**
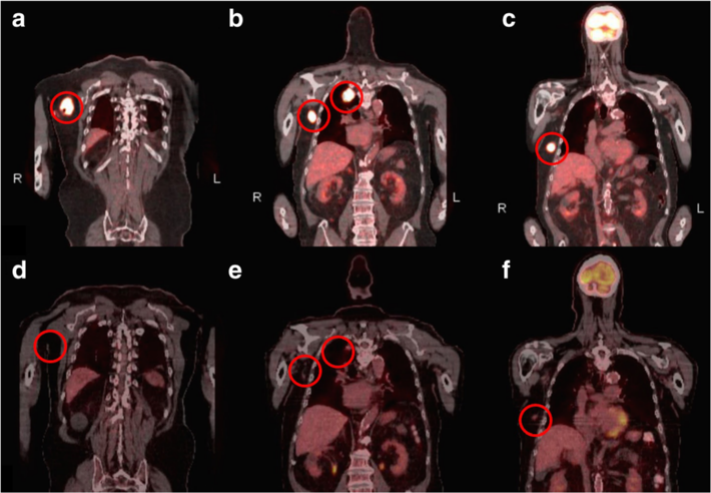
Baseline and repeat FDG-PET/CT scan illustrating areas of FDG uptake. Legend: **a**, **b** and **c** Baseline FDG-PET/CT scan revealed hypermetabolic activity consistent with metastatic disease. **d**, **e** and **f** Repeat FDG-PET/CT scan following cycle 5 of nivolumab demonstrated significant decrease in size and FDG uptake of all sites of disease

We report a case of a man in his 80s who initially noted a lesion on his right back thought to be an infected cyst in 2014. Evaluation noted right axillary adenopathy, which prompted a biopsy of the mass and of the cyst-like lesion. Pathology demonstrated morphologic and immunohistochemical (strongly cytokeratin-20, neuron specific enolase and CD56 positive and thyroid transcription factor-1 negative) findings in both lesions consistent with MCC. Polymerase chain reaction performed on deoxyribonucleic acid extracted from formalin-fixed, paraffin-embedded tumor tissue alongside appropriate controls using a previously described protocol demonstrated that the tumor lacked detectable Merkel cell polyomavirus (MCPyV) large T antigen and small T antigen [10, 11]. Staging fluorodeoxyglucose-positron emission tomography/computed tomography (FDG-PET/CT) revealed lesions in right upper lung, right upper back, right axilla and right adrenal gland consistent with metastatic disease (Fig. 1a, b and c). Subsequent biopsy of the right lung confirmed metastatic MCC. Following extensive discussions regarding the implications and options, he refused chemotherapy. At that time, no clinical trial was available to him, but programmed death-1 (PD-1) inhibitor therapy was being tested on trial (ClinicalTrials.gov. NCT02267603). Nivolumab, a humanized IgG4 monoclonal anti-PD-1 antibody, was obtained from Bristol-Myers-Squibb outside of a clinical trial, and he was subsequently treated with nivolumab 3 mg/kg intravenously every 2 weeks for six cycles in 2015. Following two cycles, he had an excellent partial response on physical exam with decreased adenopathy. After five cycles, he achieved a marked partial metabolic response by FDG-PET/CT (Fig. 1d, e and f). He continued on therapy, but after cycle six, treatment was complicated by pneumonia and autoimmune hepatitis. He improved on intravenous steroids at an outside hospital and was discharged on prednisone 1 mg/kg per day. He completely recovered with a slow steroid taper, and no further treatment with nivolumab was given. He continued on surveillance alone for 8 months, and his disease remained well controlled with no recurrence on physical exam and at least an excellent partial response in his distant metastasis with no new sites of disease.

## Conclusions

As our understanding of the oncogenic pathways of MCC evolves, immunotherapy has become a rational and promising treatment option in the advanced or metastatic setting. In 2008, Feng et al. identified a clonally integrated polyomavirus (MCPyV) in the majority (85 %) of human MCCs [12]. Although the oncogenic potential of MCPyV is still being elucidated, the increased risk of MCC in the setting of immunosuppression supports its viral-mediated origin. Circulating antibodies to MCPyV T antigen and MCPyV-specific CD8 and CD4 T-cells have been identified in patients with MCC, but appear unable to eradicate MCPyV positive cells despite immune activation. Several lines of evidence support the expression of programmed death-1/programmed death-ligand 1 (PD-1/PD-L1) as a likely contributor to immune evasion in MCC [13, 14]. Lipson et al. analyzed 67 specimens from 49 patients for PD-L1 expression by immunohistochemistry and demonstrated tumor cells and immune infiltrates expressed PD-L1 (49 % and 55 %, respectively) with 97 % of PD-L1-expressing MCC cells geographically associated with immune infiltrates [15]. Afanasiev et al. reported a higher expression of MCPyV specific circulating T-cells with PD-1 expression compared to control Epstein-Barr virus and cytomegalovirus specific T-cells (*p* < 0.01) [16]. They also found that blocking PD-1 led to augmentation of MCPyV specific T-cell function [16]. Additionally, tumors with high PD-L1 expression were more likely to have CD8 lymphocyte infiltration than tumors with lower PD-L1 expression [16]. Other investigators demonstrated that 50 % of non-activated T cells expressed PD-1, which is thought to be a marker of T cell exhaustion [17]. Taken together, these data strongly support the PD-1/PD-L1 inhibitory axis as an immune evasion strategy for MCC, and targeting this negative signal of T-cell activation could be an important treatment approach against MCC.

**Fig. 2:**
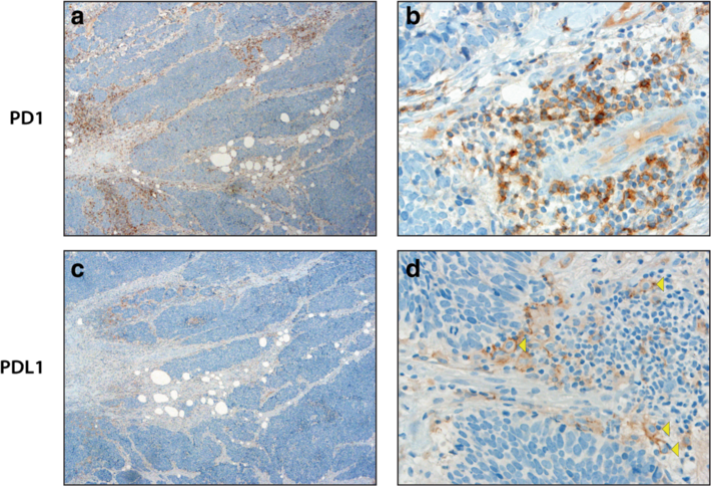
PD-1 and PD-L1 expression in Merkel cell carcinoma primary tumor from the right upper scapula. Legend: **a**, **b**: PD-1 shows patchy expression in tumor-associated lymphocytes. **c**, **d**: There is no significant PD-L1 expression in tumor cells. Background inflammatory cells express patchy PD-L1, predominantly in larger cells consistent with histiocytes (*yellow arrowheads*). Magnification 40x (**a**, **c**) or 400x (**b**, **d**)

Immunohistochemistry performed retrospectively on the tumor excised from the patient’s back demonstrated variable expression of PD-1 in tumor-associated lymphocytes (Fig. 2a and b). PD-L1 testing demonstrated patchy staining of tumor-associated inflammatory cells, predominantly histiocytes (Fig. 2c and d). There was no significant expression of PD-L1 in MCC tumor cells in comparison to positive controls. This finding is consistent with other cancers treated with PD-1 inhibitors in that PD-L1 expression may not predict the benefit from these agents [18]. Mutational load was not examined in this case. However, based on recent genomic findings in MCPyV-negative tumors, we would predict this MCPyV-negative case to display a high mutational burden with associated neoantigens that may result in susceptibility to immunotherapy [11, 19].

Preliminary results of two phase II trials provide additional evidence of PD-1 and PD-L1 inhibition as a valid and promising therapeutic approach [20, 21]. Nghiem and colleagues demonstrated that pembrolizumab (a PD-1 inhibitor) has a 56 % overall response rate in chemotherapy naïve patients with response durations ranging from 2.2 months to at least 9.7 months [20]. Avelumab (a PD-L1 inhibitor) was tested in patients with chemotherapy-refractory MCC and was shown to have a response rate of 31.7 %, with 82 % of respondents having ongoing responses at a median follow-up of 10.4 months [21]. This case highlights the potential therapeutic benefit of nivolumab, including durability of response with immunotherapy for MCC. A clinical trial evaluating nivolumab as a treatment option for virus-associated cancers, including MCC, is currently being conducted (ClinicalTrials.gov. NCT02155647).

## Abbreviations

CT: Computed tomography
FDA: Food and Drug Administration
FDG: Fluorodeoxyglucose
IgG: Immunoglobulin G
kg: Kilogram
MCC: Merkel cell carcinoma
MCPyV: Merkel cell polyomavirus
mg: Milligram
PD: Programmed death
PD-L1: programmed death-ligand 1
PET: Positron emission tomography
