# Erratum to: Metastatic Merkel cell carcinoma response to nivolumab

**DOI:** 10.1186/s40425-017-0221-x

**Published:** 2017-03-06

**Authors:** Frances M. Walocko, Benjamin Y. Scheier, Paul W. Harms, Leslie A. Fecher, Christopher D. Lao

**Affiliations:** 1 University of Michigan Medical School, Ann Arbor, MI USA; 2 Division of Hematology/Oncology, Department of Internal Medicine, University of Michigan, C451 Med Inn, 1500 East Medical Center Drive, Ann Arbor, MI 48109 USA; 3 Department of Pathology, University of Michigan, Ann Arbor, MI USA; 4 Department of Dermatology, University of Michigan, Ann Arbor, MI USA

## Erratum

Following publication of this article [1], it was noticed that the Clinical Trial Number in the last paragraph of the conclusions section was incorrectly listed as NCT02155647. The correct Clinical Trials Number is NCT02488759.
